# Multiple genetic loci influence vaccine-induced protection against *Mycobacterium tuberculosis* in genetically diverse mice

**DOI:** 10.1371/journal.ppat.1012069

**Published:** 2024-03-07

**Authors:** Sherry L. Kurtz, Richard E. Baker, Frederick J. Boehm, Chelsea C. Lehman, Lara R. Mittereder, Hamda Khan, Amy P. Rossi, Daniel M. Gatti, Gillian Beamer, Christopher M. Sassetti, Karen L. Elkins

**Affiliations:** 1 Center for Biologics Evaluation and Research, Food and Drug Administration, Silver Spring, Maryland, United States of America; 2 Department of Microbiology and Physiological Systems, UMass Chan Medical School, Worcester, Massachusetts, United States of America; 3 Department of Internal Medicine, University of Michigan, Ann Arbor, Michigan, United States of America; 4 College of Medicine, University of Cincinatti, Cincinatti, Ohio, United States of America; 5 The Jackson Laboratory, Bar Harbor, Maine, United States of America; 6 Texas Biomedical Research Institute, San Antonio, Texas, United States of America; New Jersey Medical School, UNITED STATES

## Abstract

*Mycobacterium tuberculosis* (M.tb.) infection leads to over 1.5 million deaths annually, despite widespread vaccination with BCG at birth. Causes for the ongoing tuberculosis endemic are complex and include the failure of BCG to protect many against progressive pulmonary disease. Host genetics is one of the known factors implicated in susceptibility to primary tuberculosis, but less is known about the role that host genetics plays in controlling host responses to vaccination against M.tb. Here, we addressed this gap by utilizing Diversity Outbred (DO) mice as a small animal model to query genetic drivers of vaccine-induced protection against M.tb. DO mice are a highly genetically and phenotypically diverse outbred population that is well suited for fine genetic mapping. Similar to outcomes in people, our previous studies demonstrated that DO mice have a wide range of disease outcomes following BCG vaccination and M.tb. challenge. In the current study, we used a large population of BCG-vaccinated/M.tb.-challenged mice to perform quantitative trait loci mapping of complex infection traits; these included lung and spleen M.tb. burdens, as well as lung cytokines measured at necropsy. We found sixteen chromosomal loci associated with complex infection traits and cytokine production. QTL associated with bacterial burdens included a region encoding major histocompatibility antigens that are known to affect susceptibility to tuberculosis, supporting validity of the approach. Most of the other QTL represent novel associations with immune responses to M.tb. and novel pathways of cytokine regulation. Most importantly, we discovered that protection induced by BCG is a multigenic trait, in which genetic loci harboring functionally-distinct candidate genes influence different aspects of immune responses that are crucial collectively for successful protection. These data provide exciting new avenues to explore and exploit in developing new vaccines against M.tb.

## Author summary

The genetic makeup of people contributes to their susceptibility to infection and to their ability to be protected by vaccines. This is the case with *Mycobacterium tuberculosis* and the lung disease caused by this bacterium, known as tuberculosis, where host genetics plays an important role in susceptibility to infection. However, less is known about genetic control of whether vaccination can protect people against developing tuberculosis. We used a small animal model called Diversity Outbred mice to study genetic control of successful vaccination with *M*. *bovis BCG*, the only tuberculosis vaccine used worldwide. We found sixteen gene regions associated with different outcomes after vaccination, such as how vaccination impacted lung bacterial burdens, systemic bacterial burdens, or weight loss. Importantly, the results imply that successful vaccination results from the combined activities of multiple genes which affect different features of immune responses.

## Introduction

*Mycobacterium tuberculosis* has infected humans for over 8,000 years, and during that time humans and M.tb. have likely influenced the genetic evolution of one another [1–3]. People infected with M.tb. exhibit a variety of outcomes, ranging from bacterial clearance to subclinical or latent infection, pulmonary tuberculosis (TB), or disseminated disease [4]. Several factors are known to drive heterogeneity in TB disease manifestations, including the environment, M.tb. bacterial strain variations, and the underlying susceptibility of the host (as reviewed in [5]). Adding to the complicated disease picture is the impact of *M*. *bovis* BCG vaccination on infection: BCG has been administered to over 3 billion people [6], and a wide spectrum of TB disease outcomes is also seen in vaccinated people. BCG’s efficacy against pulmonary disease in adults is quite variable and also influenced by many factors, which have been discussed in detail elsewhere [6]. Nonetheless, infants continue to be vaccinated with BCG at birth in many parts of the world because BCG clearly protects children against disseminated M.tb. infection [6]. This divergence in protection against disseminated versus pulmonary TB also illustrates the complexities in defining vaccine-induced “protection” against tuberculosis: vaccination may impact some, but not all, aspects of TB disease presentation.

Host genetics clearly contributes to resistance to primary mycobacterial infections. For example, Mendelian susceptibility to mycobacterial disease (MSMD) arises from rare human inborn genetic errors that result in susceptibility to mycobacteria normally of low virulence [7]. Defects in IFN-γ, IL-12/23, and IRF8-controlled immune pathways were identified using genetic linkage analyses in MDSM patients [8–12]. Moreover, mice with targeted deficiencies in IFN-γ, IL-12, and IRF8 have increased susceptibility following M.tb. infection [13–15]. The gene NRAMP1 was linked to host susceptibility to mycobacteria first in mice and subsequently linked to susceptibility to tuberculosis in people, illustrating the potential relevance of mice for human genetic studies [16]. However, such discrete mutations account for only a very small fraction of human TB infections. In contrast, data from a large human study that examined TB incidence rates in twins suggested high heritability for TB susceptibility, implying that TB control in most people may be complex and multigenic; however, this interpretation has been challenged [17,18].

Until recently, mouse models could not approach the genetic diversity found in humans and non-human primates. The Collaborative Cross (CC) and Diversity Outbred (DO) mice were developed to improve this situation. CC and DO mice began with the interbreeding of eight founder strains, comprising five inbred laboratory strains (A/J, C57BL/6, 129, NOD, and NZO) and three strains derived from wild caught mice representing geographically different genetic mouse clades (PWK, WSB, and CAST) [19]. The eight founder strains were placed into eight-way breeding funnels to generate many inbred mouse lines, resulting in CC mice with high natural genetic diversity. In parallel, DO mice were generated by successive outbreeding and are now highly heterogeneous, with sequence diversity approximating that of humans. DO mice are particularly well suited for fine genetic mapping and have been used to map host genetic loci associated with phenotypic traits, susceptibility to viral and bacterial pathogens, and responses to cancer therapeutics [20,21].

Investigators have mapped Mtb-related QTL in both CC and DO mice. Smith *et al*. first demonstrated that the eight CC/DO founder strains and two of the CC lines had heterogenous responses to both M.tb. primary challenge and to M.tb. challenge following BCG vaccination [22]. Of particular note, the CC/DO founder strains that were the most susceptible to primary infection were not necessarily the strains that were the least protected by BCG [22]. These results were extended to identify chromosomal regions involved in control of primary M.tb. infection, as well as genetic loci that interact with the pathogen itself [23]. Lai *et al*. recently demonstrated variable protection against M.tb. across a large panel of BCG-vaccinated CC lines, in some cases via non-canonical (non-Th1) mechanisms [24]. We studied BCG vaccination in DO mice and demonstrated that, unlike inbred laboratory mice, the population exhibited a very wide range of outcomes following M.tb. aerosol challenge, from minimal respiratory or systemic disease to fulminant disease and death [25].

Taken together, results to date indicate that genetic control of primary M.tb. infection and protection induced by BCG vaccination may be driven by non-overlapping factors [22,25]. Moreover, while previous studies in CC and DO mice support an important role for genetic background in BCG efficacy, it remains unclear whether the various traits that encompass “efficacy” are commonly controlled by the same genetic loci, or whether protection is a multigenic trait in which many loci control different facets of protective immune responses. Therefore, we pursued genetic mapping studies in DO mice to characterize the genetic landscape that controls BCG-induced protection against M.tb. infection. Using a large cohort of DO animals and multiparameter profiling, we discovered that BCG efficacy is a multigenic trait, in which distinct genetic loci harboring functionally-distinct candidate gene functions influence different aspects of protective immune responses. This insight is critical to understanding, and improving, tuberculosis vaccine efficacy in diverse populations.

## Results and discussion

### Different aspects of protection against M.tb. are under independent genetic control

To examine genetic contributions to vaccine-induced protection against M.tb., we studied a sufficient number of DO mice to power genetic mapping studies [20]. Initial studies were performed with male mice, but due to aggression issues with males, the bulk of the studies were performed with female mice. Therefore, we vaccinated ~ 1000 female and ~ 100 male DO mice with BCG intradermally and challenged the mice aerogenically with M.tb. eight weeks after vaccination. Mice were followed for survival through 14 weeks after challenge, and all surviving animals were then euthanized and tissues were collected for CFU enumeration, evaluation of lung histopathology, and genetic analyses.

**Fig 1:**
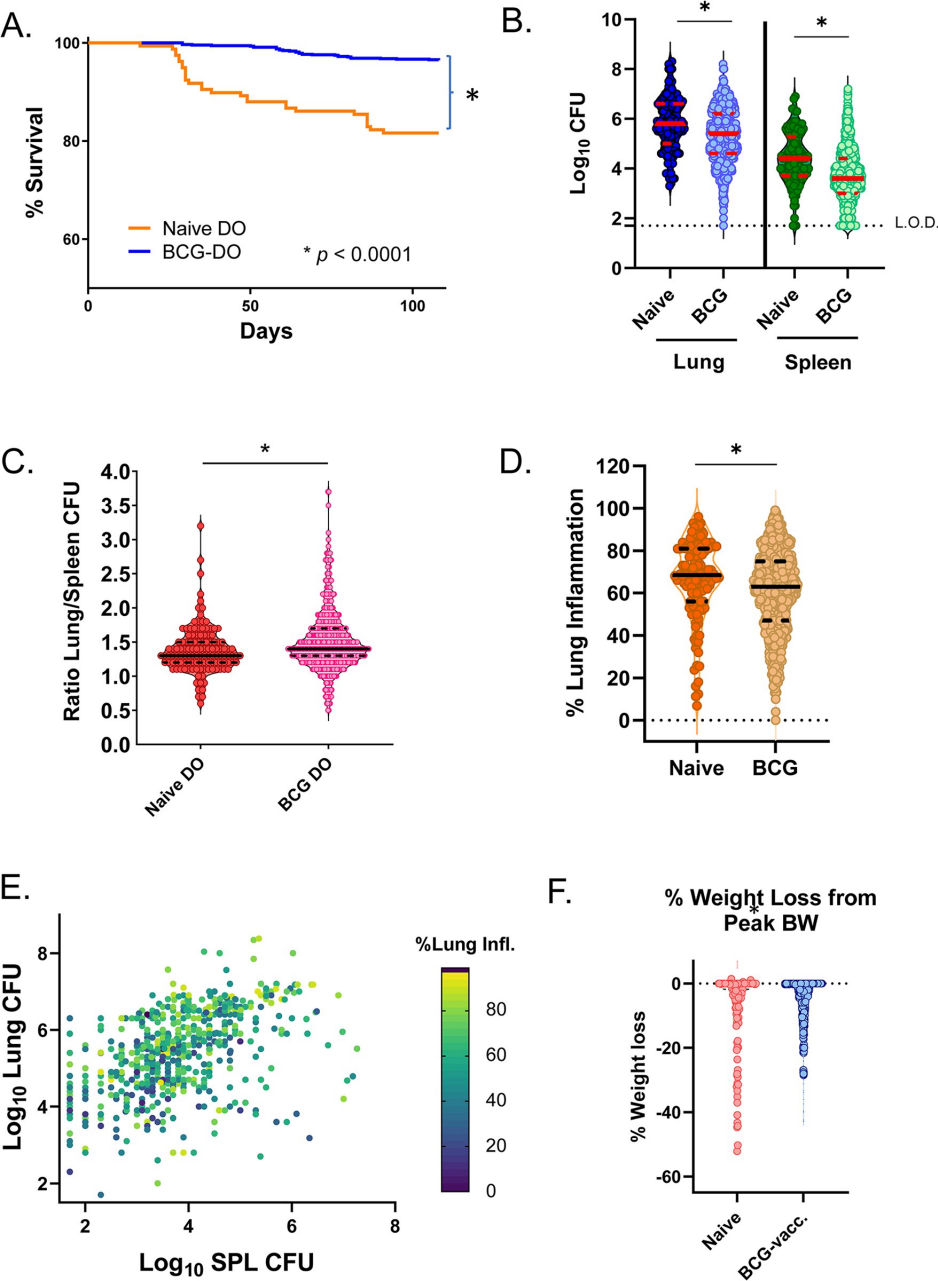
BCG vaccination followed by M.tb. challenge of DO mice leads to heterogenous infection outcomes by 14 weeks. ~ 871 DO mice were vaccinated with 10^5^ BCG Pasteur intradermally and challenged 8 weeks after vaccination with ~ 50 CFU M.tb. Erdman, and M.tb. burdens in organs assessed at 14 weeks after challenged (BCG). ~ 135 sham-PBS vaccinated DO mice were included as controls for M.tb. infection (naïve). At necropsy, a lung lobe was collected from each mouse, formalin-fixed, H&E stained, and analyzed by densitometry to assess the proportion of inflamed lung tissue. (A) Survival of M.tb.-challenged naïve and BCG-vaccinated DO mice through 14 weeks after challenge (*p < 0.0001; Kaplan-Meier). (B) Lung and spleen M.tb. burdens for naïve and BCG-vaccinated DO mice. (C) The ratio of the lung CFU/spleen CFU which was determined for each of the naïve-M.tb. challenged or BCG-vaccinated/M.tb.-challenged DO mice. (D) The percent of inflamed lung tissue for each mouse from naïve or BCG-vaccinated mice. Groups were compared by *t*-test, *p < 0.0001. (E) The lung and spleen CFU and percent lung inflammation are represented for each BCG-vaccinated/M.tb.-challenged mouse, with the percentage of lung containing inflammation depicted by color scale. (F) Body weight was tracked over time for all naïve and BCG-vaccinated mice that were challenged with M.tb. For each animal, the percent of weight loss from the peak body weight was calculated; individual animals are represented by dots. For (B), (C), (D), and (F) data are depicted as violin plots, with individual mice represented as dots. Median value for each group is represented by a solid line, with quartiles represented by dashed lines. Groups were compared by Student’s *t* test, *p < 0.05.

We previously published the outcomes for an initial set of ~ 250 of these DO mice [25]. Consistent with our previous observations, BCG vaccination improved survival after challenge in the ~ 1000 mouse mapping population, in terms of the proportion of mice that succumbed to infection and an extended time to death for those mice with early morbidity (*p* < 0.0001; Kaplan-Meier) (Fig 1A). BCG protected the DO population by reducing the mean lung and spleen M.tb. burdens compared to those of the naïve DO group, although bacterial burdens were quite heterogeneous (*p* < 0.0001, *t*-test) (Fig 1B). M.tb. burdens in lungs and spleens ranged over 10,000-fold between individual animals, from almost 10^9^ colony forming units (CFU) per organ to undetectable (< 1.7 log_10_ CFU), a level of bacterial control rarely observed in inbred mice. We also observed a BCG-induced shift in the ratio of CFU in the lungs compared to CFU in spleens toward a more pulmonary-skewed disease, likely due to a greater number of vaccinated-DO mice with undetectable splenic M.tb. (Fig 1C). This observation is reminiscent of the ability of BCG to protect against disseminated or miliary TB in children, but not pulmonary TB in adults.

We measured the percentage of lung tissue with inflammation in these animals by applying quantitative densitometry to histopathology slides (Fig 1D). The lungs of many DO mice contained a high proportion of tissue with inflammation and disease involvement at this late time point. However, a protective effect was observed in the BCG-vaccinated mice, with a mean reduction of 5% in inflammation across the populations (*p* < 0.0001, *t*-test). We then directly compared lung CFU, spleen CFU, and lung inflammation across the BCG-vaccinated, M.tb.-challenged DO mice (Fig 1E). This view of the combined traits reiterated that, across the population, all possible combinations of lung CFU, spleen CFU, and lung inflammation were observed, supporting the conclusion that these traits may be under independent genetic control. Weight loss is a typical hallmark of advanced tuberculosis in humans, yet relatively few DO mice lost substantial weight following challenge (Fig 1F). However, BCG did provide moderate protection against weight loss, determined as a percentage weight loss from peak (*p* < 0.0001, *t*-test) (S1C Fig). Given the moderate phenotype associated with BCG-induced protection, weight loss traits such as body weight at euthanasia and the percent of peak body weight at euthanasia were also included in genetic mapping studies.

**Fig 2:**
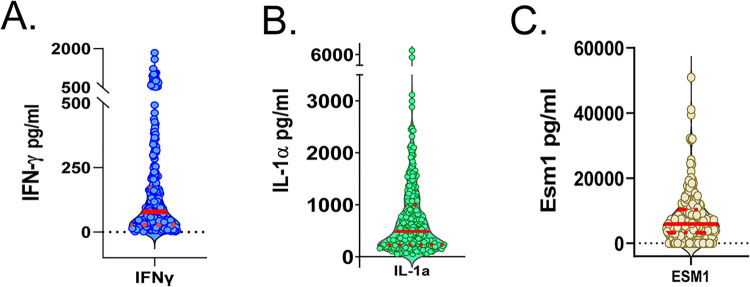
Lung homogenates from BCG-vaccinated/M.tb.-challenged DO mice contain variable amounts of cytokines at 14 weeks after challenge. Lung homogenates from a subset of 300 BCG-vaccinated/M.tb.-challenged mice were analyzed for the presence of a panel of 37 chemokines and cytokines by multiplex assay or sandwich ELISA (S1 Table). Data are presented for three of the cytokines, (A) IFN-γ, (B) IL-1α, and (C) Esm1. Data are represented by violin plots, where individual mice are represented as dots, the median value for each group by a solid red line, and quartiles by dashed red lines.

In order to better understand inflammatory activities and host immune responses in the lungs of BCG-vaccinated/M.tb.-challenged DO mice, we measured a panel of 37 chemokines and cytokines in lung homogenates (S1 Table). Lungs from a random subset of 300 BCG-vaccinated animals collected at necropsy 14 weeks after M.tb. infection were evaluated. Cytokine expression was heterogeneous across the DO population for all cytokines tested (S1 File). For example, concentrations of the Th1 cytokine IFN-γ ranged over 2,000-fold between individual animals (Fig 2A). Other cytokines such as IL-1α (Fig 2B) and Esm1 (Fig 2C) were similarly variable, with concentrations ranging from 7,000–60,000-fold from animal to animal.

**Fig 3:**
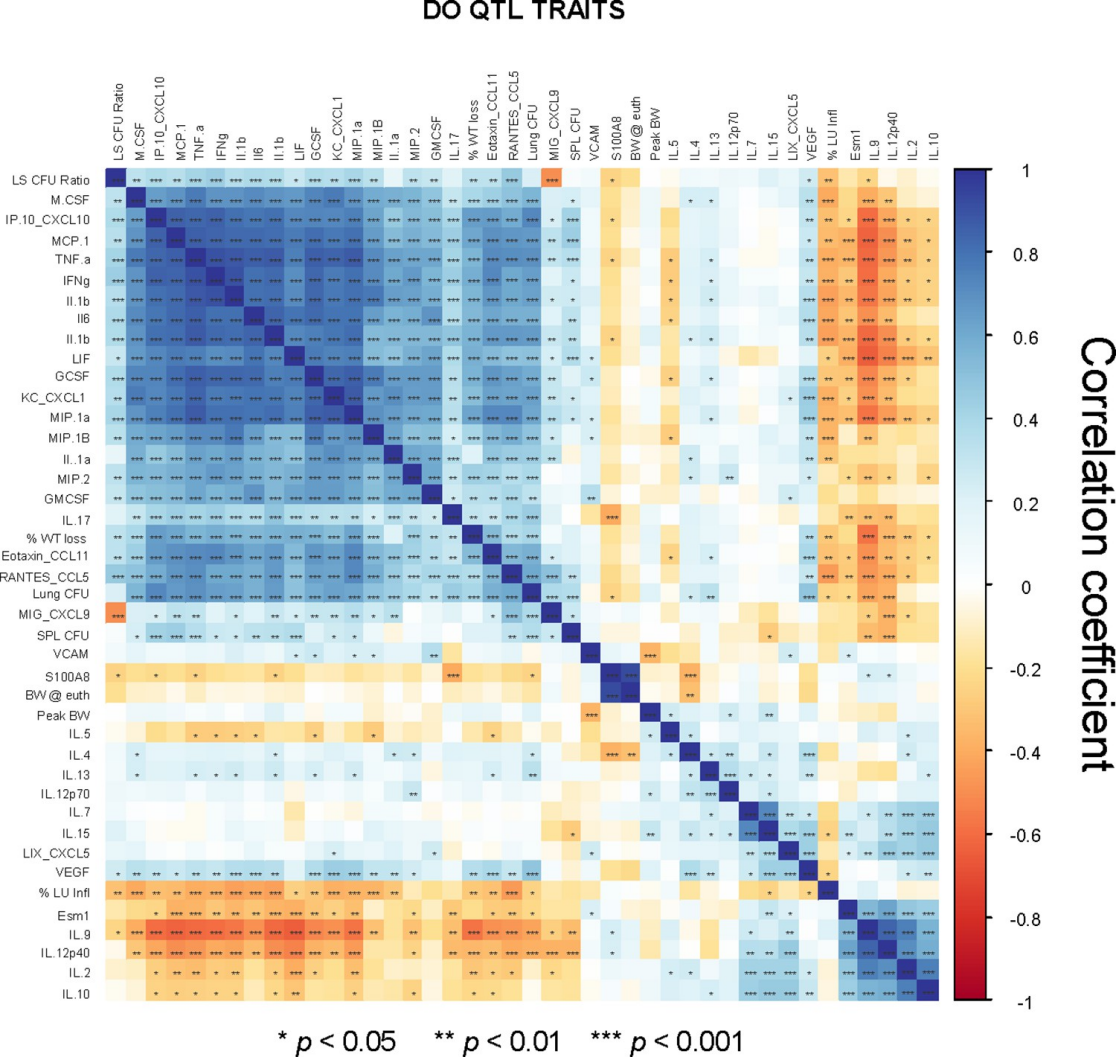
Pearson’s correlations reveal associations between complex disease traits and lung cytokine content. Data from all complex and cytokine traits for BCG-vaccinated/M.tb. challenged DO mice collected at 14 weeks after challenge of vaccinated mice were used to determine Pearson’s correlations between each trait. Data were analyzed using R studio, ggplot package. Color scale represents the strength of the Pearson’s *r* coefficient from -1 (red; negative correlation) to 1 (blue, positive correlation). Asterisks represent those with significant associations; *p*-values are represented by *p < 0.05, ** *p* < 0.01, ***p < 0.001.

We compared the associations between lung cytokines and complex infection traits (lung and spleen CFU, lung inflammation, and weight traits) using Pearson’s correlation analyses (Fig 3 and S2 Table). Lung CFU numbers positively correlated with spleen CFU (Pearson’s r = 0.510, *p* = 8.48e^-7^) and weakly negatively correlated with lung inflammation (Pearson’s r = -0.102, *p* = 1.93e^-5^). As previously observed [25], weight loss was not significantly associated with lung or spleen M.tb. burdens, nor with lung inflammation (*p* > 0.3). Several cytokines had significant positive correlations with lung CFU, including pro-inflammatory cytokines such as TNF-α and IL-6 (Fig 3). These cytokines may either be driving the progression of disease and/or reflect the degree of M.tb. burdens. We examined traits with negative correlations with M.tb. organ burdens because these could be potential correlates of protection. IL-9, IL-12 p40, and Esm1 had the strongest negative correlations with lung and spleen M.tb. However, the relatively weak correlations that we observed further support the interpretation that these traits are independently controlled.

### Multiple genetic loci control distinct vaccine-related complex infection traits

Taken together, measurements of the complex infection traits and of lung cytokine levels present a multi-factorial snapshot of complex M.tb. disease presentations following BCG vaccination/challenge of DO mice. Therefore, we incorporated all complex infection traits and lung cytokine measurements into genetic linkage analyses (S1 Table). ~ 1000 female and ~ 100 male BCG-vaccinated/M.tb.-challenged DO mice were genotyped by GigaMUGA array at ~ 140,000 SNP positions. This information was used to perform quantitative trait loci (QTL) mapping, which identifies a section of DNA (locus) that correlates with variation of a quantitative trait. The results are reported as a logarithm of odds (LOD) score, which is an estimate of the probability that two loci, or a chromosomal locus and a trait, are linked (Table 1). QTL mapping revealed sixteen loci associated with complex or cytokine traits that passed the significant (*p* < 0.05) or suggestive (*p* < 0.2) thresholds. We named these loci *Vip* for *V*accine-*i*nduced *p*rotection. Of the complex infection traits, lung CFU, spleen CFU, lung/spleen CFU ratio, body weight at euthanasia, and weight loss mapped to fourteen loci across six chromosomes (Table 1). Traits that co-located to the same peak with overlapping confidence intervals were grouped into a single QTL region. For each QTL, we also calculated the effect size on phenotypic variation, reported as the percent variance explained (Table 1).

**Table 1:** Quantitative trait loci are associated with vaccine-induced protection against M.tb. Each QTL that reached a significant or suggestive cutoff was annotated as a *v*accine-*i*nduced *p*rotection, “*Vip*”, locus. Traits with QTL with overlapping confidence intervals and similar peak positions were grouped into a presumed putative shared QTL. Cytokine QTL are shaded in grey. Values in bold are those that were significant, *p* < 0.05. ^a^ Pos.; the position on the chromosome for the QTL peak, in Mb. ^b^ LOD; the logarithm of odds (LOD) value determined at each QTL peak. ^c^ CI; the confidence interval (CI) for each QTL region, in Mb. ^d^ % variance describes the total variance that is attributable to genetic differences. ^e^ Weight loss was calculated as the percent weight loss between body weight at euthanasia/body weight at peak.

Locus	Trait	Chr.	^a^ Pos.	^b^LOD	p-value	^c^CI Low (Mb)	CI High (Mb)	Allele Effects- High	Allele Effects- Low	^d^ % variance
***Vip1***	^e^ % Weight loss	1	73.9811	6.9324	0.16	73.2222	144.71	CAST		4.2393
***Vip2***	IL-13	1	130.68	7.1977	0.13	129.611	132.296	PWK/CAST	129/AJ/NZO/NOD/B6	12.9501
***Vip3***	BW @ euthanasia	1	149.088	6.9306	0.16	143.681	152.432		PWK	4.2325
***Vip4***	VEGF	2	29.6035	6.9464	0.19	28.8645	30.4636	NOD	CAST	11.3712
***Vip5***	MIP.2	5	33.421	6.9391	0.19	32.2072	39.339	CAST	NOD/B6	11.3601
***Vip6***	LS CFU Ratio	6	8.65405	7.5485	0.06	8.15516	53.4413		WSB	4.9271
***Vip7***	CCL5 (RANTES)	7	66.9833	7.1483	0.14	66.0615	68.6566	NZO	AJ/WSB	11.6817
***Vip8***	ESM1	8	37.9935	7.2484	0.11	36.6356	40.6294	PWK/WSB	AJ/NOD	13.3472
***Vip9***	LU/SPL CFU ratio	8	119.231	7.1114	0.13	117.22	119.422	NOD	PWK/B6	4.6486
***Vip10***	% Weight loss	9	88.102	7.3016	0.09	87.0912	88.9793	PWK	B6	4.4599
***Vip11***	CCL5 (RANTES)	11	83.1754	7.0310	0.16	82.0127	85.8644	NOD	CAST	11.5014
***Vip12***	IL-1a	14	**73.0295**	**7.7411**	**0.05**	**72.8579**	**73.4656**	NZO	NOD/CAST/WSB	11.8423
	CXCL1 (KC)	14	73.0473	7.2530	0.13	72.8579	74.647	NZO	NOD	12.6314
***Vip13***	LS CFU Ratio	15	73.9882	7.3920	0.08	72.5187	76.107	CAST/PWK		4.8275
	LS CFU Ratio	15	75.9	7.1978	0.11	72.5187	76.107	CAST/PWK		4.7037
	Spleen CFU	15	76.3202	6.8367	0.18	72.4589	81.3119	AJ/NZO/WSB	CAST/129	4.4166
	Spleen CFU	15	78.3184	7.4245	0.07	72.4589	81.3119	AJ/NZO/WSB	CAST/129	4.7871
***Vip14***	GMCSF	16	85.4728	7.0742	0.15	57.7981	85.8167	WSB/NOD	129	11.6923
***Vip15***	Spleen CFU	17	**33.824**	**7.5988**	**0.05**	**33.5614**	**45.9545**	PWK	B6	4.8967
	Lung CFU	17	34.4141	6.9250	0.16	32.1797	48.1432	PWK/WSB	129/B6	4.1521
	Lung CFU	17	**43.4093**	**8.2880**	**0.02**	**32.1797**	**48.1432**	PWK	B6	4.9488
	Lung CFU	17	**45.6942**	**7.6513**	**0.05**	**32.1797**	**48.1432**	PWK	B6	4.5775
	Spleen CFU	17	**45.8084**	**7.8390**	**0.03**	**33.5614**	**45.9545**	PWK		5.0475
***Vip16***	MIP-2	X	**56.4881**	**7.8366**	**0.04**	**55.8833**	**59.331**	129/WSB	NZO	12.7318
	MIP.1B	X	58.4324	6.9642	0.19	55.8833	73.9989	129	CAST/AJ/NZO	11.9947

**Fig 4:**
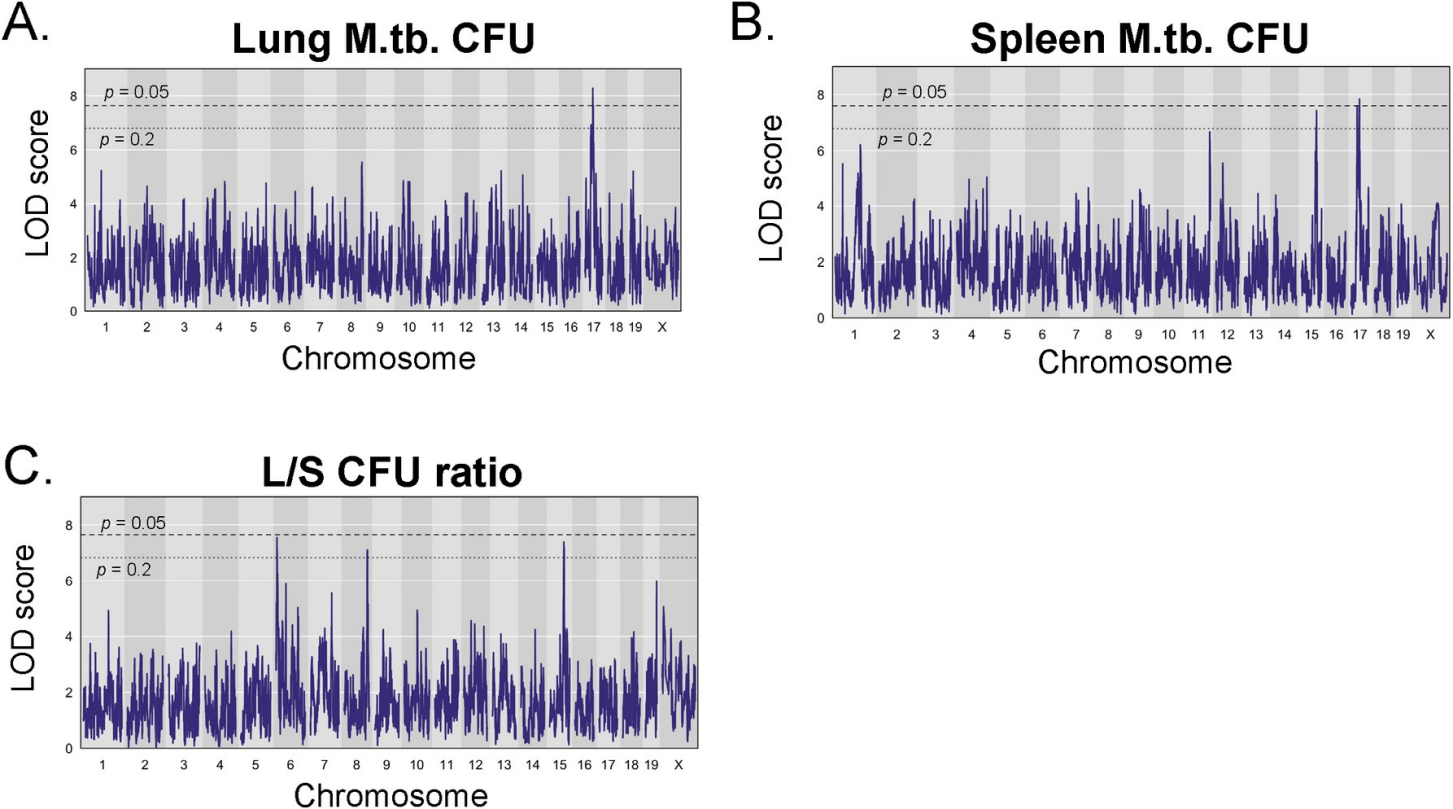
QTL mapping reveals novel QTL associated with complex outcomes following BCG vaccination and M.tb. challenge. Genome wide QTL scans were performed for the complex traits A) Lung CFU, B) Spleen CFU, and C) the Lung/Spleen CFU ratio. QTL plots represent the region of the chromosome (x-axis) and the LOD score, interpreted as the strength of the association between the trait and a particular region of the chromosome (y-axis). Dashed and dotted lines indicate P value thresholds of 0.05 and 0.2, respectively.

The highest LOD peaks for complex infection traits were two peaks on chromosome (Chr.) 17 for lung CFU (LOD 8.3, LOD 7.7) and spleen CFU (LOD 7.8), each trait having a maximum at 33 Mb and 45 Mb, respectively (Table 1 and Fig 4A and 4B). We tentatively considered them to be separate QTL, but to test this hypothesis we used the R package qtl2pleio to examine the likely existence of separate QTL versus a single, overlapping QTL affecting both traits (pleiotropy) [26]. The best fit for the data was found to be a single QTL located at about 32 Mb; evidence for separate QTL was not significant (*p* = 0.37). Therefore, this region was named *Vip15* as a single QTL. This region overlaps with the region encoding the mouse major histocompatibility (MHC) locus, which is located around 34–38 Mb [27,28]. MHC is a central component of adaptive immunity to *M*. *tuberculosis*, by which antigen-specific CD4^+^ T cells are primed via the presentation of mycobacterial peptides presented by MHC-class II [29–31]. In humans (human leukocyte antigen-HLA) and in mice, the MHC locus and specific MHC alleles are associated with increased risk of tuberculosis infection [32–40]. Finding a known region for adaptive immunity to M.tb. in this study provides validation of the mapping approach.

**Fig 5:**
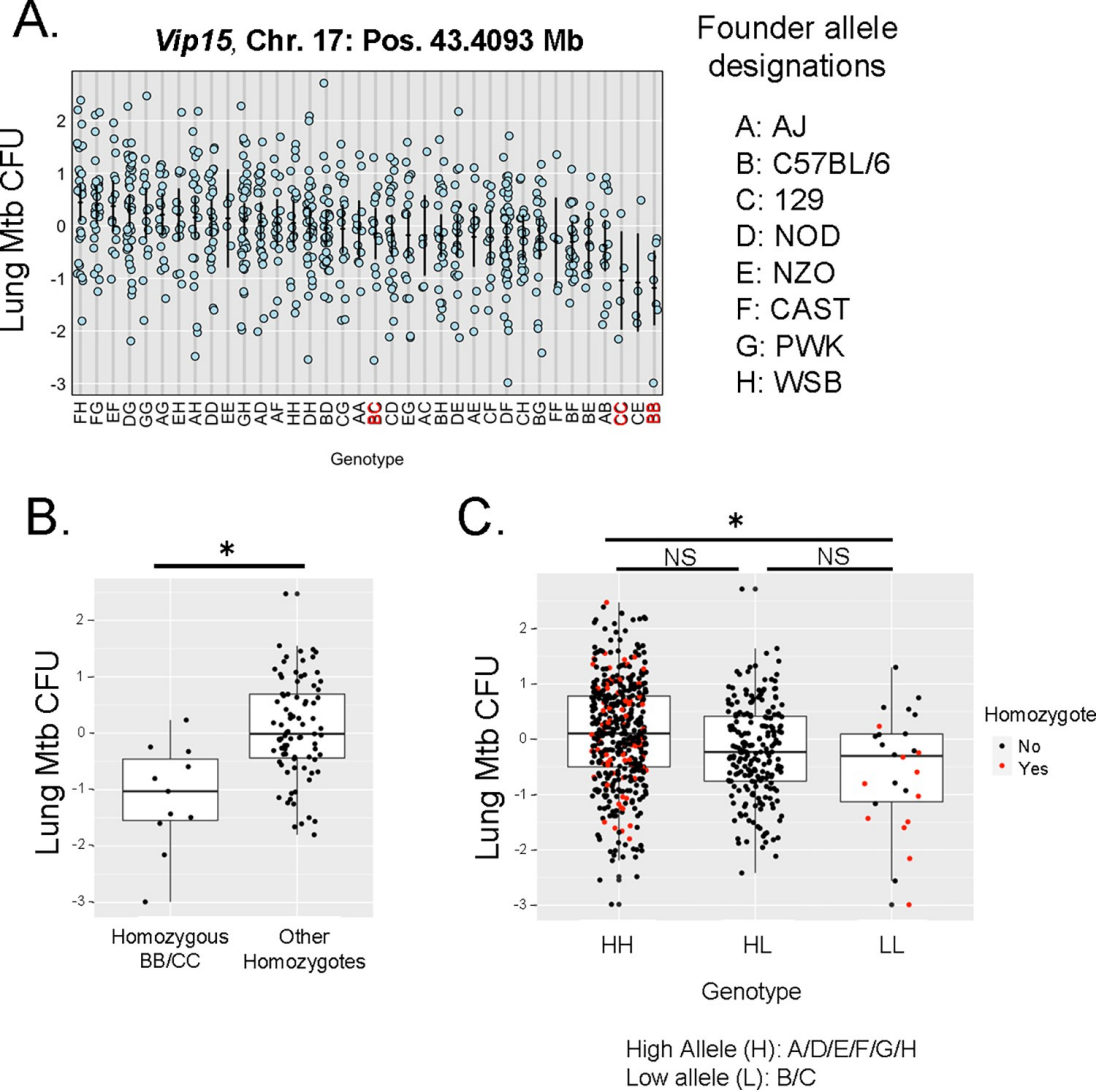
Genotype analyses infer that the *Vip15* QTL driving control of lung CFU is biallelic and dominant. Lung CFU data for each animal in the mapping study were Z-scale transformed, and founder alleles were determined for each mouse at chromosome 17 position 44.3093, the QTL peak. Alleles contributed by each of the eight founder strains are designated A: AJ, B: C57BL/6, C: 129, D: NOD, E: NZO, F: CAST, G: PWK, H: WSB. A) Individual mice are represented by dots, plotted according to lung CFU and the founder allele genotype at the QTL peak. B) Animals were separated in two populations that were either homozygous for the low allele (BB or CC) or the high alleles (Other Homozygotes: AA/DD/EE/FF/GG/HH). The two groups were compared by Welch’s two sample *t*-test and were found to be significantly different, * *p* < 0.05. C) Animals were separated into three populations based on whether they possessed a high (H) allele (A/D/E/F/G/H) or low allele (L) (B/C) at the peak position. Populations representing mice homozygous for a high allele (HH), homozygous for a low allele (LL), or heterozygous with a mix of high and low alleles (HL) were compared for differences in variance by an F-test. Variance was significantly different, and therefore groups were further compared in a pairwise fashion by Welch’s two sample *t*-tests, * *p* < 0.05, or NS = p > 0.05. Animals that were homozygous for a given founder allele are represented by red dots; heterozygous animals by black dots.

To further understand the genetic drivers of M.tb. control in this region, we examined contributing allele effects by performing phenotype x genotype (PxG) analyses. Lung CFU data were plotted against the genotypes of individual mice at position 43.4 Mb on Chr. 17 (*Vip15*) (Fig 5A). Animals that were homozygous or heterozygous for B6 or 129 alleles at this locus (BB, CC, or BC alleles) exhibited lower lung CFU. We next compared mice separated into bi-allelic groups as either being homozygous for B6 or 129 alleles (BB or CC) or homozygous for one of the other alleles (Fig 5B). Mice that were homozygous for B6 or 129 had significantly lower lung CFU than the other homozygous mice, suggesting that this is a bi-allelic trait (*p* < 0.05, by Welch’s two sample *t*-test). We further compared animals that were homozygous for a high allele (HH), homozygous for a low allele (LL), or heterozygous for a high/low allele (HL). We found no significant differences between animals that were HL and animals that were LL homozygous, or between HL animals and those that were HH homozygous (*p* < 0.05, by Welch’s two sample *t*-test). Although not significant, the HL animals did have an intermediate phenotype; this suggests that the low/protective allele may be dominant, but further studies will be necessary to evaluate this possibility (Fig 5C). We also note that B6 and 129 mice carry the same MHC haplotype, and so these results are consistent with previous studies in which H-2 haplotype was associated with resistance to M.tb. [31]. Also of note, the MHC allele distribution across the 8 founder strains is complex, and for several of the founder strains is unpublished; there are likely 7 MHC alleles across the 8 founder strains (M. Ferris, personal communication). Future plans include assessing the contribution of MHC versus other genes to this QTL.

Another complex infection trait QTL, *Vip1*, overlaps with the region containing NRAMP or SLC11A1, which is also a chromosomal region known to control the host response to mycobacteria (Table 2). This region was originally identified in mice as containing a single dominant gene that directs host control of several intracellular infections, including *M*.*bovis BCG*, *Salmonella typhimurium*, and *Leishmania donovani* [41,42]. In mice and in humans, *SLC11A1* variants are associated with autoimmune disorders as well as increased susceptibility to infectious diseases [42]. In addition to NRAMP, this region also contains the genes for *cxcr1* and *cxcr2*, which are also associated with disease susceptibility; further, expression of these genes is induced by BCG vaccination [43–45]. Discovering a QTL in this region further supports the validity of the approach and the resulting findings.

**Table 2:** Candidate genes for each *Vip* QTL. The top gene candidates were determined for each QTL region. Table 2 contains the size of the region contained within the confidence interval for each QTL (in Mb) and the number of annotated protein coding genes within that region. Cytokine QTL appear in grey. For QTL encompassing multiple peaks, the overlapping confidence interval between peaks was analyzed. High interest gene candidates are those protein coding genes within the region containing SNPs with high LODs. For CCL5, a QTL mapped to a region containing the protein coding gene for CCL5 in bold. ^a^ CI = confidence interval; ^b^ NRAMP/Slc11a1 and the MHC regions are known to influence host susceptibility to microbial pathogens including *M*. *tuberculosis*. ^c^ Most open reading frames in this region are unannotated.

Locus	Trait	Chr.	QTL CI (Mb)	# of genes within QTL ^a^CI	Top gene candidates
***Vip1***	% Weight loss	1	71.5	465	Tns1, Arpc2, Cxcr2, Rufy4, Pnkd, Arpc2, Cxcr1, ^b^ Slc11a1, Ctdsp1
***Vip2***	IL-13	1	2.7	38	Fcamr, Zp3r, Pigr, C4bp, CD55b, Rab29, Cdk18, Lemd1, Thsd7b
***Vip3***	BW @ euthanasia	1	8.8	32	^c^ Pla2g4a, Hmcn1
***Vip4***	VEGF	2	1.8	39	Med27, Ntng2, Setx, Ttf1, Rapgef1, Ak8, Cfap77, Barhl1, Ddx31, Gtf3c4
***Vip5***	MIP.2	5	7.1	87	Ppm1g, Adra2c, Dok7, Cpz, Htra3, Plk-ps1, Trmt44, Acox3, Zbtb49
***Vip6***	LS CFU Ratio	6	45.3	321	Ica1, Umad1, Mios, Col28a1, C1galt1
***Vip7***	CCL5 (RANTES)	7	2.6	19	Selenos, Lrrk, Fam189a1, Pcsk6, Synm, Ttc23, Chsy1, Snrpa1
***Vip8***	ESM1	8	4.0	13	Sgcz, Mfhas1, Zdhhc2, Dlc1, Tusc3
***Vip9***	LS CFU Ratio	8	2.2	8	Cdh13, Mlycd
***Vip10***	% Weight loss	9	1.9	14	Zfp949, Trim43a, Mthfsl, Tbx18, Nt5e
***Vip11***	CCL5 (RANTES)	11	3.9	64	**CCL5**, CCL4, Hnf1b, Heatr6, Asic2, Wfdc21, Bcas3, Ppm1d
***Vip12***	IL-1a	14	1.8	5	Cysltr2, Rcbtb2, Rb1, Lpar6, Itm2b
	CXCL1 (KC)	14	0.6		
***Vip13***	LS CFU Ratio	15	3.6	63	Col22a1, Dennd3, Ptp4a3, Ptk2, Ago2, Trappc9, Chrac1, Kcnk9, Zc3h3, Gsdmd, Mroh6, Eef1d
	LS CFU Ratio	15	3.6		
	Spleen CFU	15	8.9		
	Spleen CFU	15	8.9		
***Vip14***	GMCSF	16	28.0	81	App, Jam2, Cyyr1
***Vip15***	Spleen CFU	17	12.4	293	Kank3, Cd320, Kifc1, 2-Ob, Btnl1, ^b^H2-Ab1, ^b^H2-Aa, ^b^H2-Eb2, ^b^H2-DMb1, Neu1, Adgrf5, RunX2, Supt3, Clic5
	Lung CFU	17	16.0		
	Lung CFU	17	16.0		
	Lung CFU	17	16.0		
	Spleen CFU	17	12.4		
***Vip16***	MIP-2	X	18.1	26	Arhgef6, Zic3, Fgf13
	MIP.1B	X	3.4		

**Bold** = The coding region for CCL5 is located within the confidence interval for *Vip11*

Three QTL for lung/spleen CFU ratio mapped to chromosomes 6 (*Vip6*), 8 (*Vip9*), and 15 (*Vip13*; Fig 4C and Table 1). Interestingly, only one of the lung/spleen CFU ratio QTL, *Vip13*, overlapped with a locus for spleen CFU, while none overlapped with lung CFU loci. Body weight is a complex trait regulated by multiple QTL even in naïve animals (as reviewed in [46]). Finally, we found that body weight at euthanasia (S1A Fig) and the percent of weight loss from peak body weight after BCG-vaccination/M.tb. infection (S1B Fig) mapped to two separate chromosomes, Chr. 1 and 9, both of which are novel loci for associations with weight (Table 1). Whether these QTL associations with weight are specific for weight during M.tb. infection or weight in general are not yet known.

### Multiple genetic loci control distinct vaccine-related cytokine traits

**Fig 6:**
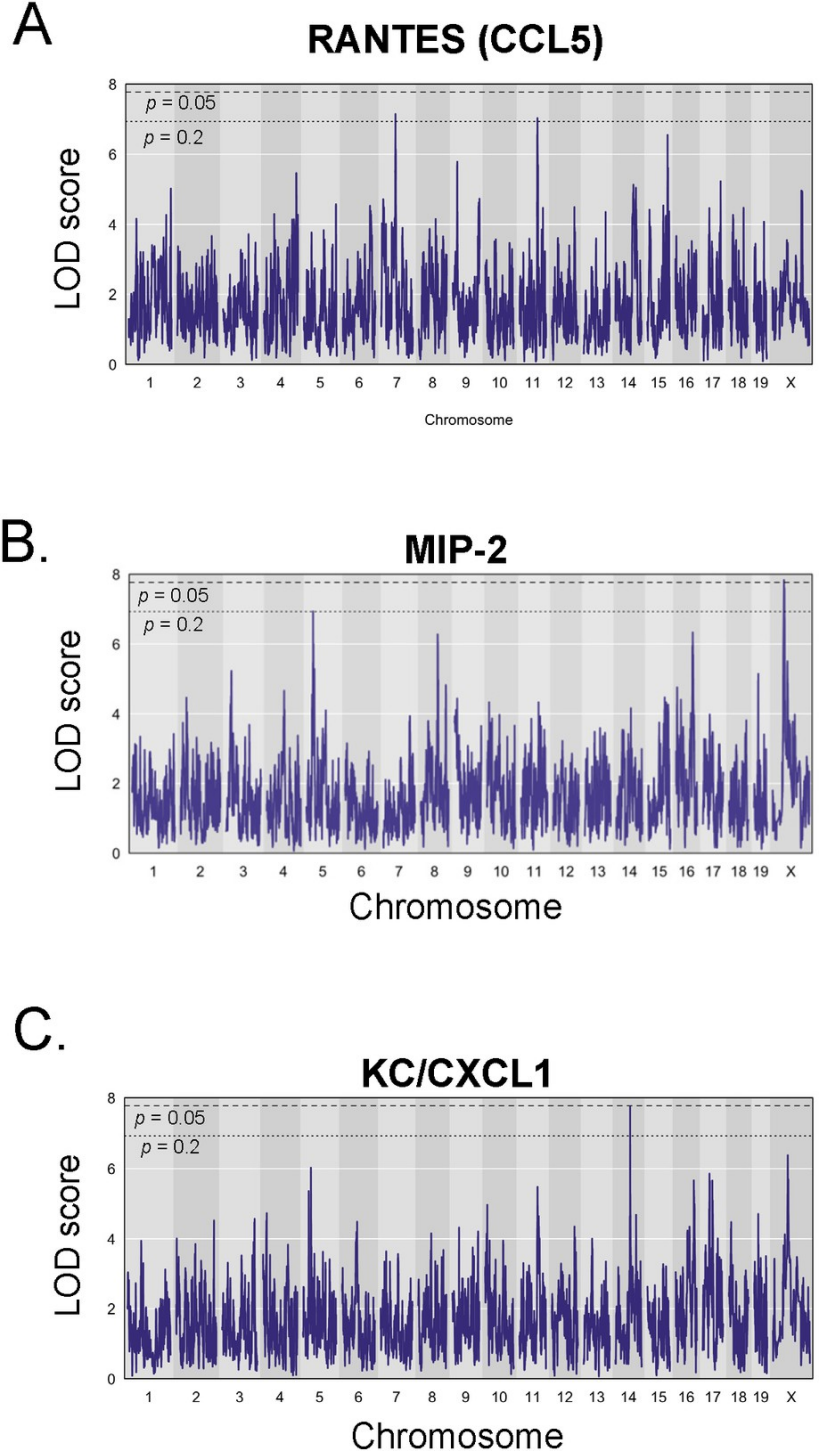
QTL mapping reveals novel QTL associated with lung cytokines following BCG vaccination and M.tb. challenge. Data from 37 cytokines and chemokines derived from 300 BCG-vaccinated/M.tb.-challenged DO mice were used to perform QTL mapping. Nine cytokines had significant or suggestive QTL, of which three are presented here: A) RANTES/CCL5, B) MIP-2, C) CXCL1. A threshold of *p* < 0.05 for significant (dashed line) or *p* < 0.2 for suggestive (dotted line) traits was set for the analyses.

Using the same cutoff criteria for significant or suggestive loci, we also found QTL associations for nine cytokines across ten chromosomes (Table 1). Of all the cytokine QTL, only one overlapped with the coding region for the cytokine itself, CCL5/RANTES on chromosome 11 (*Vip11*; Fig 6A). *Vip7* on chromosome 7 was also defined by lung RANTES/CCL5 expression (Table 1 and Fig 6A). The *Vip7* QTL region overlaps with several known QTL related to TB host responses derived in animals, including *Tip1* [47], *Tbrs5* [48], Trl-3 [49] and a QTL linked to human TB susceptibility [50]. RANTES/CCL5 is a chemokine that influences cell migration, cell activation, and the development of Th1 immune responses via signaling through a panel of receptors, including CCR5. While the role of RANTES itself has not been studied in the context of primary M.tb. infection, CCR5 was demonstrated to influence cell migration into lungs and lymph nodes after M.tb. challenge [51,52]. The roles of neither CCR5 nor RANTES itself have been investigated in the context of vaccine-induced protection against M.tb., representing potential areas for future studies.

The cytokine QTL with the highest statistical significance was for Mip-2 on the X chromosome (*Vip16*; Fig 6B). A QTL for MIP-1β also mapped to the same region at 58.43 Mb (S2A Fig), suggesting that these represent a common QTL. Lung MIP-1β and MIP-2 levels were also correlated by Pearson’s correlation analyses (Fig 3; r = 0.653, p = 2.14e^-11^), further supporting a common regulatory element within this region for both cytokines. All animals included in the cytokine analyses were female, which raises the question of whether sex-linked effects contribute to relative cytokine expression. This possibility will be addressed in future studies. A second, suggestive QTL for Mip-2 mapped to Chr. 5 (*Vip5*, LOD 6.93, Table 1). A suggestive QTL for IL-13 (*Vip2*) mapped near *Vip3*, a QTL for body weight at euthanasia (S2C Fig). However, the confidence intervals for *Vip2* and *Vip3* do not overlap. Thus, a common regulatory element for both traits may not be present in this region. In fact, we found no direct overlap between any cytokine QTL and any of the QTL for complex traits. This is not necessarily surprising, as several biological steps are likely to be between a regulatory element influencing cytokine production and the ultimate biological outcome of a disease trait such as lung CFU. Such a scenario would result in independent chromosomal regions influencing a given cytokine and a disease outcome. Additional suggestive QTL for VEGF (S2D Fig), Esm1 (S2E Fig), and GM-CSF (S2F Fig) occurred on chromosomes 2, 8, and 16, respectively (Table 1).

Overlapping significant and suggestive peaks for KC/CXCL1 (Fig 6C) and IL-1α (S2B Fig) on Chr. 14 shared a peak at ~ 73 Mb and a narrow confidence interval less than 2 Mb. Therefore, these likely represent a shared QTL driving both traits, denoted as *Vip12*. This region does not contain the coding regions for either IL-1α or CXCL1, or any known regulators of these cytokines, suggesting that this may represent a novel regulatory region for this immune pathway. IL-1α is an upstream regulator of CXCL1. Therefore, overlapping QTL for these cytokines could be an effect of regulatory control of IL-1α, which in turn affects production of CXCL1 [53]. Both IL-1 α and CXCL1 have established links to TB immunity. Studies using mice deficient in IL-1R, IL-1α, or in IL-1 α/β double deficient mice demonstrated that the IL-1/IL-1R signaling pathway plays a role in controlling M.tb. infection [54–57]. CXCL1 was previously identified as a strong correlate of M.tb. disease in DO mice, and a biomarker of disease progression in humans [58,59]. The links between IL-1α, CXCL1, and the host response to M.tb. make *Vip12* another attractive and high priority candidate for follow-up studies. In particular, the role for these host factors in the vaccine-induced response to M.tb. remain largely unstudied.

### Allele effects distinguish founder contributions to complex and cytokine QTL

**Fig 7:**
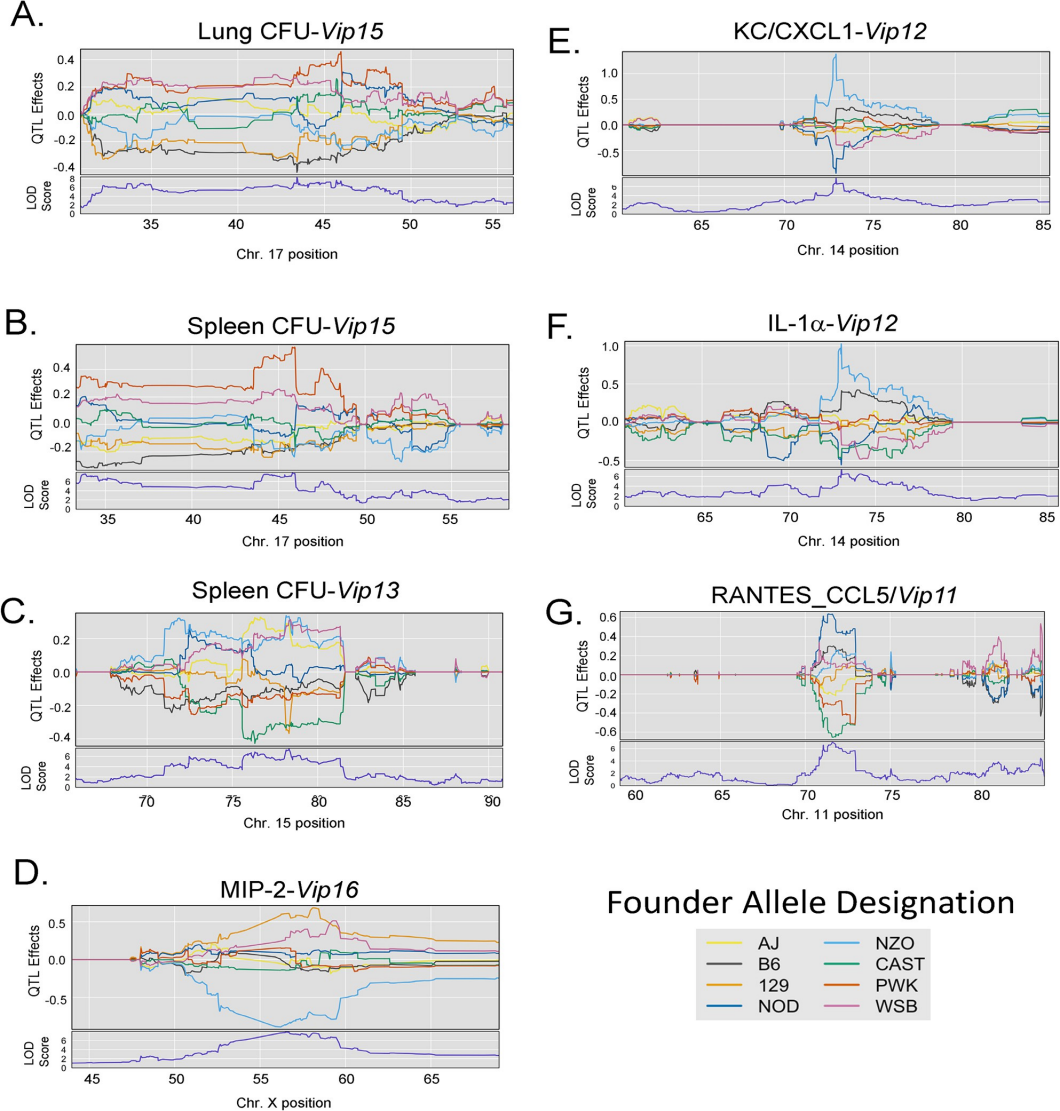
Founder allele effects for each locus demonstrate the relative genetic contributions of each founder strain to the QTL. Allele effect plots were generated using the plotting functions of the qtl2 package. Each colored line represents the allelic contribution of a given founder strain as depicted in the legend. The allele effects were determined for A) Lung CFU/*Vip15*, B) Spleen CFU/*Vip15*, C) Spleen CFU/*Vip13*, D) Mip-2/*Vip16*, E) KC/CXCL1/*Vip12*, F) IL-1α/*Vip12*, and G) RANTES/CCL5/V*ip11*.

The GigaMUGA genotyping array allows for extended genotyping analyses of DO mice. The GigaMUGA SNP probes were designed to differentiate alleles from the eight founder strains. Therefore, GigaMUGA data can be interrogated to infer the contributing founder alleles for a given chromosomal location (Table 1). The imputed founder allele contribution to a given trait (QTL effect) can be plotted for a given QTL region. For example, for *Vip15*, genetic contributions from PWK drove high lung and spleen CFU, while B6/129 alleles contributed to low CFU (Fig 7A and 7B). *Vip13* encompasses both spleen CFU and lung/spleen CFU ratio QTL. Low spleen CFU at this locus is driven by CAST/129 (Fig 7C), while a high lung/spleen CFU ratio is similarly driven by CAST/PWK (S3A Fig). This aligns with a shift in low spleen CFU driven by CAST and resulted in a higher L/S CFU ratio, also driven by CAST. PWK contributed to a high lung/spleen CFU ratio for *Vip6*, while WSB contributed low effects (S3B Fig). Founder allele effects were similarly determined for the remaining complex QTL (Table 1 and S3D–S3F Fig).

For the most significant cytokine QTL, *Vip16*, a 129 allele was associated with elevated MIP-2 (Fig 7D) and MIP-1β (Table 1 and S3A Fig). IL1-α and CXCL1, the shared traits underlying *Vip10*, had similar allele effects with strong NZO high and NOD low drivers (Fig 7E and 7F). A *cis*-QTL, one that resides near the coding region for the gene, for RANTES/CCL5 (*Vip11*) had strong NOD high and CAST/PWK low allele effects (Fig 7G). Allele effects for remaining suggestive cytokine QTL are presented in S4 Fig.

**Fig 8:**
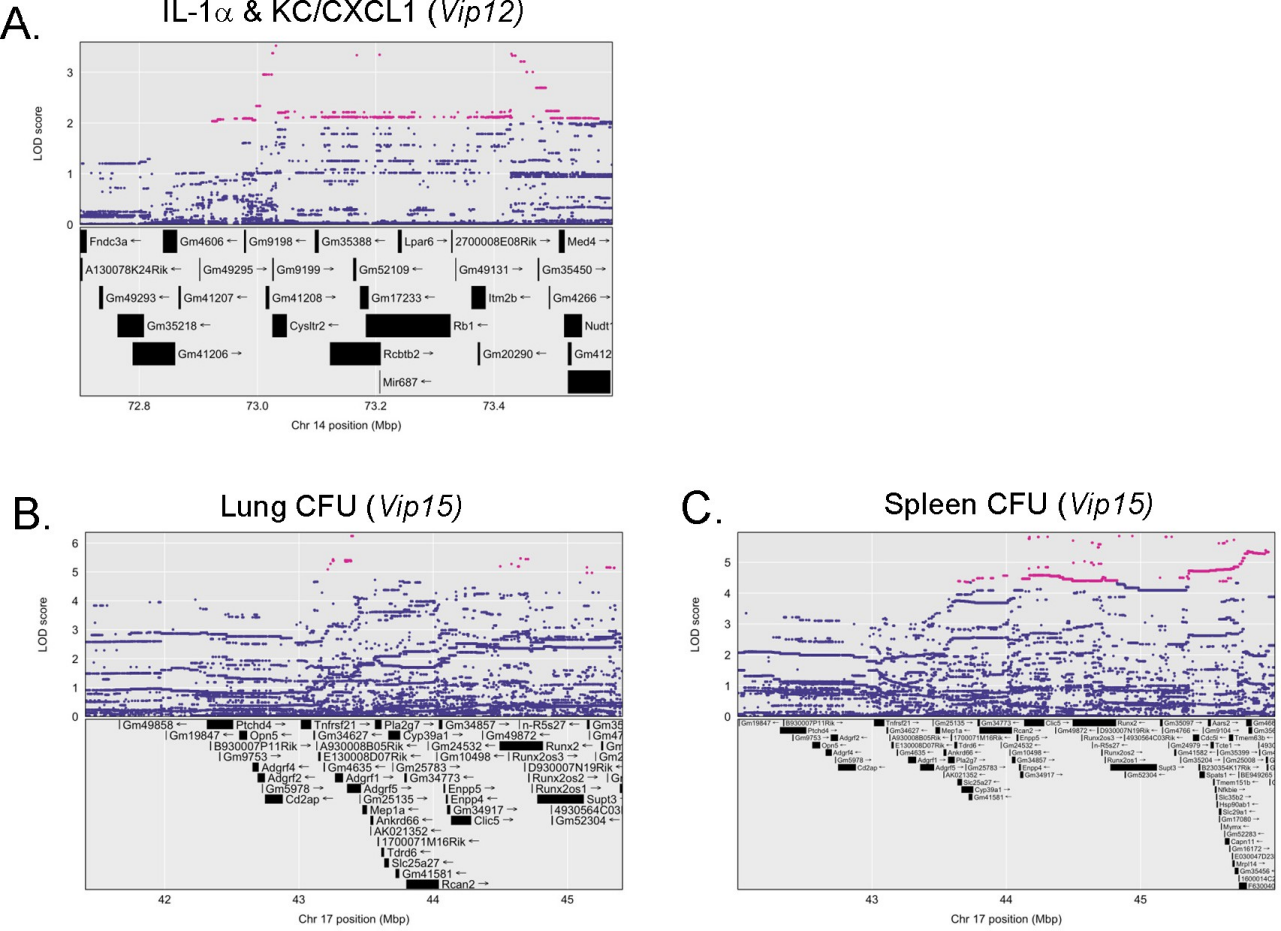
Individual single nucleotide polymorphisms (SNP) are associated with complex infection and cytokine traits. Allele probabilities were converted to SNP probabilities over selected intervals of interest and scanned for trait association. SNPs within 1.5 LOD of the maximum are plotted in pink, with SNPs below this threshold plotted in blue. Annotated coding regions for genes are listed across the chromosomal region of interest, and the chromosomal position (Mbp). SNPs are presented for A) the overlapping region for *Vip12*, and for *Vip15* the SNPs associated with B) Lung CFU and C) Spleen CFU.

Within the significant chromosomal intervals for each QTL, we used the size of the confidence interval (CI) for a given QTL to examine the number of annotated protein coding genes within that region (Table 2). For QTL covering multiple traits, we used the region of overlap between CI. We then tested for an association between individual SNPs and a given trait within the region of interest. The top gene candidates in each region were determined based on LOD score coupled with finding a strain distribution series that matches the founder allele effects for this region. Overall, five QTL had CI less than 3 Mb, with three QTL CI less than 2 Mb. Many SNPs across all QTL were located in non-coding intergenic regions. The CI for *Vip12* was the narrowest of any QTL in this study, with an overlapping region of 0.6 Mb mapped using IL-1α and CXCL1 data (Table 2). This region contains only five annotated protein coding genes, and sixteen unannotated open reading frames (Fig 8A). When we examined the allele effects for individual SNPs in coding genes, we found high LOD SNPs within *cysltr2* and *Rb1* with allelic series matching those driving the trait, while the other three genes did not.

Determining the precise interval for *Vip15* is complicated by the presence of five QTL peaks within this region that encompasses ~ 16 Mb and contains almost 300 annotated genes. As mentioned above, the *Vip15* region also contains many genes related to the MHC locus. Within the confidence interval for this QTL, we found high LOD SNPs for both lung and spleen CFU traits in several genes such as *adgrf5*, *runx2*, *supt3*, and *clic5* (Fig 8B and 8C). SNPs in these regions also have allelic series that match the founder allele effects for this region, making these high priority gene candidates for this QTL. While we cannot determine whether MHC alone is the driver of the phenotypes in the region, other attractive gene candidates that may be contributing to host responses are evident. Of particular interest is Adgrf5 (GPR116), a G-protein coupled receptor located at ~ 43Mb on chromosome 17 that is predominantly found on the surface of alveolar type I and type II cells [60]. Adgrf5 has a role in regulating pulmonary surfactant homeostasis and contributes to the regulation of alveolar macrophage activation [61–63]. Given the association of *adgrf5* within the QTL for lung CFU and the link between *adgrf5* and lung biology, this is a high priority candidate for follow-up studies.

### Summary and perspectives

Human and mouse genome-wide association studies have uncovered genetic linkages to susceptibility to M.tb. However, these studies have largely focused on the genetics that underlie resistance to primary M.tb. infection. Resistance to primary infection may not be driven by the same host factors that drive vaccine-induced protection, but host genetics that contribute to the efficacy of BCG have not been as widely studied. The recent studies by Smith *et al*. [22] and Lai *et al*. [24] support the role that underlying host genetics plays in influencing responses to BCG-vaccination. The results shown here further these findings by directly examining genetic contributions to BCG-induced protection against M.tb., as revealed in extensively outbred DO mice that are uniquely suited to genetic mapping.

Using a large population of BCG-vaccinated/M.tb.-challenged DO mice, we found sixteen QTL associated with complex infection outcomes after infection or with cytokines produced in lungs after challenge (Table 1). Complex outcomes represented both data collected up until euthanasia, such as mouse weights over time, and data collected when the mice were euthanized at 14 weeks after challenge, such as lung CFU, spleen CFU, and lung histopathology. Taken together, the genetic analyses of a large population of DO mice revealed novel QTL associated with complex outcomes after vaccination/M.tb. challenge and with cytokines produced in lungs in response to vaccination and challenge. Of note, in parallel a very similar study has now performed QTL mapping using naïve DO mice challenged by aerosol with M.tb. only (G. Beamer, personal communication). Only one common QTL that shares overlapping confidence intervals between both studies was found. This is a QTL on Chr. 17 (called *Vip15* here, or *dots17* there). In the present vaccine-related study, this QTL is associated with lung and spleen M.tb. burdens, while in the primary infection study this QTL is associated with granuloma necrosis. Further, there is a discrepancy in founder allele effects for these QTL, and SNP and downstream analyses suggest different gene candidates in this area (none of which are MHC). Exploring the relationship between these two QTL will be a topic for future studies. Nonetheless, taken together the combined data support the interpretation that the majority of the vaccine-related QTL uncovered in this study are unique and distinct from those found in mapping studies of primary M.tb. infection. Moreover, the majority of QTL described here represent previously unappreciated linkages between these chromosomal regions and M.tb. control. Downstream analyses that determined the founder allele effects and significant SNPs for each QTL support follow-up studies, with a number of plausible candidates identified. Studies of high priority gene candidates will potentially inform studies to evaluate novel mechanisms of vaccine-induced protection, as well as potential novel pathways and regulatory elements for controlling cytokine responses during successful vaccination against TB.

Recently, Smith *et al* described novel QTL associated with control of M.tb. primary infection. These QTL were derived using CC mice and were designated *Tip* for *t*uberculosis *i*mmuno*p*henotype [47]. Interestingly, only two of the *Vip* QTL found here overlap with *Tip* QTL. *Tip3*, *Tip7*, and *Tip10* in CC mice were associated with lung IFN-γ, lung IL-17, and lung M.tb. CFU, respectively, and are all located on chromosome 15 [23,47]. These CC-derived QTL overlap with the *Vip13* locus, which was associated with spleen M.tb. and lung/spleen CFU ratios in DO mice following BCG-vaccination/M.tb.-challenge (Table 1). Allele effect determinations suggest that CAST is likely the driver of low spleen CFU as well as the shift toward an elevated lung/spleen CFU ratio (Fig 7C). In the CC mice, CAST was also associated with *Tip3* and *Tip10*. *Tip3* was validated using a cross of two CC lines, which confirmed the association between this locus and IFN-γ production following primary infection [47]. However, the relationship to vaccine-induced protection has not yet been tested for these loci. An examination of *Tip10* by CC cross did not validate the linkage between that QTL and lung M.tb. burdens, although the authors propose that the reduced complexity cross used to address the linkage did not incorporate the haplotype that represented the strongest phenotype at the locus of interest [23]. That the *Vip13* locus also maps to the same region further supports underlying genetic factors in that region that control host responses to M.tb. Further studies are warranted to investigate the importance of this chromosomal region and the specific biological mechanism driving protection, especially in the context of vaccine-induced control of M.tb.

In general, less is known about the genetic drivers of vaccine responses compared to those influencing primary immunity, and for many pathogens these immune pathways may overlap only partially. In humans, the most well-documented associations are for vaccines that generate humoral immunity and the linkage with a subset of HLA haplotypes. Certain HLA alleles have been associated with responsiveness to vaccines for viral and bacterial diseases such as SARS CoV-2, measles, mumps, influenza, hepatitis B, diphtheria, and polio based on associations with antibody production [64–69].

Non-HLA genes have also been implicated in controlling vaccines responses. For example, genetic variations in the signal-regulatory protein family SIRP were linked to persisting MenC-specific serum binding antigen (SBA) antibodies generated in response to the capsular group C meningococcal conjugate vaccine [70]. Related to the current work, a recent study found loci that regulate cytokine production in restimulated whole blood samples collected after BCG vaccination [71]. One of the SNPs found in that study that is related to TNF-α production following restimulation of the host blood with mycobacterial purified protein derivative (PPD) overlaps with a homologous region of interest in this study, *Vip6*. Another SNP from the same study was associated with IL-5 production and has a homolog downstream of *Vip15*, although *Vip15* does not directly overlap within the confidence interval of that QTL. Significant heritable associations were also found between HLA and cytokine responses to BCG vaccination in a study of twins in Gambia [69]. However, these studies are limited to associations with targeted cytokine production following cellular restimulation and are not direct linkages between any alleles and disease outcomes. We believe that a strength of the present study lies in the combination of mapping both cytokine traits and complex infection traits, providing a broad picture of genetic associations with vaccine-induced protection against M.tb.

Collectively, these results support the use of the DO mice as a tool for modeling vaccine-induced protection against tuberculosis and for interrogating the genetic basis of vaccine responses. Most of the genetic associations with vaccine-induced protection revealed in this study are unique from those associated with control of primary infection, underscoring the need to better understand the interplay of host genetics and vaccine-primed host immune responses. Altogether, we found sixteen QTL associated with specific features of vaccine-induced protection against M.tb. Moreover, the results indicate multigenic control that collectively reduced development of overt, symptomatic disease. Several of these QTL represent known factors associated with immune response to M.tb., yet few have been studied in the context of vaccination. Thus, future studies of these QTL may reveal new mechanisms by which vaccine-induced responses are controlled.

## Materials and methods

### Ethics statement

All studies utilizing animals were performed under protocol #2011–14, which was approved by the Institutional Animal Care and Use Committee (IACUC) of the Center for Biologics Evaluation and Research at the Food and Drug Administration. Animal protocols stressed practices and procedures designed to strictly minimize any suffering.

### Mice and animal care

Male and female C57BL/6J and Diversity Outbred (DO) mice were purchased from Jackson Laboratories (Bar Harbor, ME) and were used when 6–10 weeks of age. DO mice were obtained from breeding generations 20–32. Initial experiments were performed with male mice, but because some male DO mice were quite aggressive and had to be individually housed, subsequent experiments were performed with female mice. Within each experiment, all animals were age and sex matched. All mice were housed in microisolator cages (NexGen cages, Allentown LLC) with Biofresh Comfort Bedding (Scott Pharma Solutions) and were given irradiated Lab Diet 5P76 and water *ad libitum*. Mice were housed in a room with set environmental criteria at 45–55% humidity with a temperature of 72°F on a 12 hour light/dark cycle.

### Bacteria and growth conditions

*Mycobacterium bovis* BCG Pasteur (BCG) and *M*. *tuberculosis* Erdman (M.tb. Erdman) were derived from the mycobacterial culture collection of the Trudeau Institute and propagated once to prepare working seed stocks, which were then used to prepare all infection stocks without further passage. M.tb. Erdman and BCG Pasteur were grown in 7H9 media supplemented with OADC, glycerol, and 0.05% Tween 80 (Difco Laboratories, Detroit, MI) to mid-logarithmic phase as previously described, then vials for single infection use were frozen in 0.5 ml aliquots at -80°C until use [72]. A sample from each batch of bacterial stock was subjected to quality control experiments to determine the number of colony forming units (CFU), to confirm typical colony morphologies, and to confirm vaccination efficacy or infection in mice.

### BCG vaccination and *M*.*tb*. challenge

For each experiment, a single frozen vial of BCG was thawed and diluted to the desired concentration in sterile PBS. Groups of 5–10 B6 or 30–160 female DO mice were vaccinated subcutaneously with 10^5^ CFU of BCG or were sham-vaccinated with phosphate buffered saline (PBS, low endotoxin, Lonza, Walkersville, MD). Animals were challenged by aerosol with M.tb. Erdman using a Middlebrook Chamber (Glas-Col, Terra Haute, IN) over a 30-minute exposure period with and average targeted ~ 50 CFU delivered to the lungs. Within each aerosol run, a group of five C57BL/6 animals were included to determine actual aerosol uptake 4 hours after challenge, when mice were euthanized and M.tb. burdens assessed in lungs. Actual delivered dose ranged from approximately 7–73 CFU, with an average of ~ 45 CFU. Mice from eleven independent vaccination experiments of similar design were pooled to generate the data presented here, for a total of 135 naive DO mice and 871 BCG-vaccinated DO mice. Animals were weighed prior to challenge and weekly after challenge. Mice that began to show signs of illness such as ruffled fur, weight loss, or cachexia were weighed daily. Mice were monitored and euthanized when clearly unable to reach food and water, following pre-established humane endpoint criteria. Biological data from an initial set of ~ 250 mice included in the current genetic mapping study were previously published [73].

### Assessment of bacterial organ burdens and tissue pathology

Bacterial burdens in organs were determined at 14 weeks after infection, or when a mouse met criteria for humane euthanasia before 14 weeks. Mice were euthanized, and organs were removed aseptically and transferred to sterile homogenizer bags containing 5 ml of sterile PBS per organ. Organs were disrupted using a Stomacher (Seward, England), and the homogenates were serially diluted and plated for CFU enumeration on 7H11 plates containing 10% OADC enrichment (Becton Dickinson, Sparks, MD) medium, 10 μg/ml ampicillin, 50 μg/ml cycloheximide, and 2 μg/ml 2-thiophenecarboxylic acid hydrazide (TCH) (Sigma). The addition of TCH to the agar plates inhibits BCG growth but has no effect on *M*. *tuberculosis* growth [74]. Organ homogenates were also frozen and stored at -80°C to be used for later analyses. In some experiments, portions of each lung and spleen were removed, lungs were inflated by delivering 10% formalin using a 27-gauge needle, and lung and spleen tissue were preserved in 10% formalin. Formalin fixed samples were then sent to American Histolabs, Inc. (Gaithersburg, MD), where the tissues were embedded in paraffin, sectioned at 5 μm, and stained with hematoxylin and eosin (H&E). The Image-Pro Plus software (Media Cybernetics, Rockville, MD) was utilized to assess the level of inflammation present in densitometry scans of each H&E-stained image. Lung inflammation was quantitated by assigning areas with dark pink and purple color staining as inflamed [75]. The percentage of dark pink and purple colored areas (compared to light pink and open areas) from a lung section of each mouse was determined by the software and reported as percent lung inflammation per sample.

### Measuring cytokines in lung homogenates

A subset of 300 mice representing the highest and lowest lung and spleen M.tb. burdens at 14 weeks after vaccination/challenge were used for cytokine analyses. 400 μl samples of lung homogenate collected at the 14 week necropsy were purified by centrifugation and double-filtration through 0.2 μm filters. Samples of filtered lung homogenate were submitted to Eve Technologies (Calgary, Canada) for cytokine evaluation using the Mouse Cytokine 32-Plex multiplex assay. Esm1, VCAM1, S100A8, and Gzmk levels were evaluated by sandwich ELISA using reagents purchased from R&D Systems (Minneapolis, MN), according to the manufacturer’s instructions. The absorbance was read at 405 nm on a VersaMax tunable microplate reader with a reference wavelength of 630 nm (Molecular Devices, Sunnyvale, CA). Cytokine concentrations were determined by comparing unknown values to a standard curve made with recombinant protein at known concentrations provided with the ELISA kit, using four-parameter fit regression in the SOFTmax Pro ELISA analysis software (Molecular Devices).

### QTL mapping methods

A tail snip from each DO mouse was collected and stored at -20°C. Samples were submitted to Neogen (Lincoln, NE) for genomic DNA purification and genoptying. Mice were genotyped using the Illumina Mouse Universal Genotyping Array (GigaMUGA) with 143,259 markers [76]. The genotyping results were reformatted for input to the R package qtl2 [77] using data files and R scripts available at https://kbroman.org/qtl2/pages/prep_do_data.html. We followed recommended procedures as described by Broman *et al* [78] for quality assurance and quality control for the DO genotype data. Samples with greater than 5% missing genotype calls were removed, as were individual markers having an estimated error rate greater than 5%. Six pairs of samples had identical genotypes and were presumed to be mislabeled. All were removed, as were six mice predicted to be chromosomally XO on the basis of X and Y chromosome marker intensities. The final mapping panel consisted of 742 mice genotyped on 111,982 markers. Using these markers, the DO genotypes, and the DO founder genotypes, the genome of each DO mouse was reconstructed in terms of 36-state haplotype probabilities, further simplified to 8-state haplotype probabilities (for the 8 CC founder strains) using qtl2.

Phenotypes were measured in 13 batches incorporating as many as three separate infection runs per batch. Rather than including each batch/run combination as an individual additive covariate in the QTL scans, batch variation was treated as a random variable, nesting run within batch. For each phenotype, a linear mixed effects model was fit using the R package lme4 [79] and the residuals were used as phenotype values for mapping. Prior to model fitting, the phenotype values were transformed using an inverse normal transformation. Cytokine traits were measured in only a subset of the female mice, 265 of which were members of the final mapping panel. Since most of these mice were derived from only a single infection run of a given batch, the individual batch/run combinations were treated as the random effect. Otherwise, data transformation and lme4 modeling was the same as for the spleen, lung, and weight loss traits.

QTL mapping was carried out using a linear mixed model with LOCO (leave one chromosome out) kinship matrices. For spleen, lung, and weight loss traits, sex was included as a covariate; other traits were measured in only females. Significance thresholds were measured by fitting generalized extreme value distributions of 5000 random permutation maxima for each trait, from which genome-wide permutation p-values were calculated. LOD profiles and allele effect plots (as BLUPs) were generated using the plotting functions of the qtl2 package. SNP association scans were performed using the scan1snps function of qtl2. QTL effect sizes were calculated using standard linear model methodologies [20]. All supporting meta data and mapping files are available: https://figshare.com/s/19b5f33b7e5496f3f42e.

### Statistical analyses

The statistical significance of differences within biological parameters was assessed using Student’s *t* test, the Mann-Whitney sum rank test, Pearson’s correlation, or others as described in the text (GraphPad Prism, San Diego, CA).

## Supporting information

S1 Fig QTL mapping reveals novel QTL associated with complex outcomes following BCG vaccination and M.tb. challenge. Genome-wide QTL scans were performed for the complex traits including A) Body weight at euthanasia, B) Weight Loss. Dashed and dotted lines indicate P value thresholds of 0.05 and 0.2, respectively.
(TIF)

S2 Fig QTL mapping reveals novel QTL associated with lung cytokine content following BCG vaccination and M.tb. challenge. Data from 37 cytokines and chemokines, derived from 300 BCG-vaccinated/M.tb. challenged DO mice, were used to perform QTL mapping. Nine cytokines had significant or suggestive QTL, of which six are presented here: A) MIP-1b, B) IL-1a, C) IL-13, D) VEGF, E) Esm1, and F) GM-CSF. A threshold of *p* < 0.05 for significant (dashed line) or *p* < 0.2 suggestive (dotted line) traits was set for the analyses.
(TIF)

S3 Fig Founder allele effects for complex trait-associated loci demonstrate the relative genetic contributions of each founder strain to the QTL. Allele effect plots were generated using the plotting functions of the qtl2 package. Each colored line represents the allelic contribution of a given founder strain, as depicted in the legend. The allele effects were determined for A) Lung/Spleen CFU ratio/*Vip13*, B) Lung/Spleen CFU ratio/*Vip6*, C) Lung/Spleen CFU ratio/*Vip9*, D) Body weight at euthanasia/*Vip3*, E) Weight loss/*Vip1*, F) Weight loss/*Vip10*.
(TIF)

S4 Fig Founder allele effects for cytokine-associated loci demonstrate the relative genetic contributions of each founder strain to the QTL. Allele effect plots were generated using the plotting functions of the qtl2 package. Each colored line represents the allelic contribution of a given founder strain as depicted in the legend. The allele effects were determined for A) MIP-1β/*Vip16*, B) IL-13/*Vip2*, C) VEGF/*Vip4*, D) MIP-2/*Vip5*, E) Esm1/*Vip8*, F) RANTES/CCL5/*Vip7*, G) GM-CSF*/Vip14*.
(TIF)

S1 Table Complex infection and cytokine traits used for QTL mapping. Data from eight complex traits related to outcomes after BCG-vaccination/M.tb. challenge were incorporated into QTL mapping studies. A panel of 37 cytokines and chemokines were quantified in lung homogenates after M.tb. challenge from a subset of 300 BCG-vaccinated DO mice, and these data were used for QTL mapping.
(XLSX)

S2 Table Pearson correlation coefficients and *p*-values for complex infection and cytokine traits from DO mapping studies. Combined complex traits and lung cytokine data were subjected to Pearson correlation analyses. The correlation coefficients (r) and *p* values (*p*) for each combination are provided.
(XLSX)

S1 File Metadata and genotype files. (XLSX)
