# Supplementary figures and images for: Multiple genetic loci influence vaccine-induced protection against Mycobacterium tuberculosis in genetically diverse mice
Source: PLoS Pathog. 2024 Mar 7;20(3):e1012069. doi: 10.1371/journal.ppat.1012069 (PMC10950258; doi:10.1371/journal.ppat.1012069)

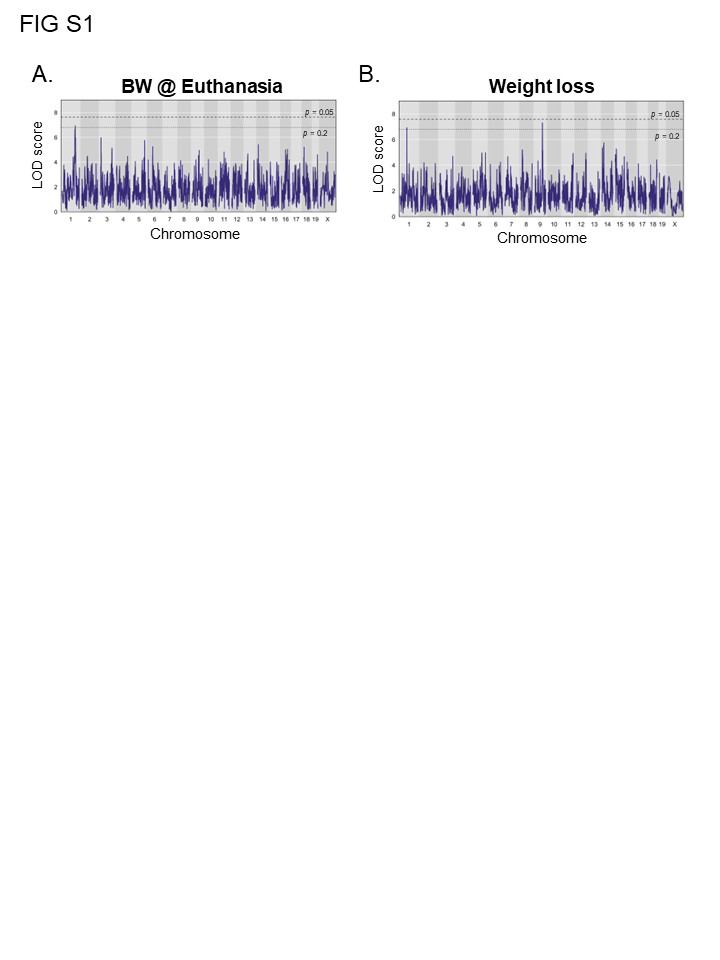

Supplement: S1 Fig — Genome-wide QTL scans were performed for the complex traits including A) Body weight at euthanasia, B) Weight Loss. Dashed and dotted lines indicate P value thresholds of 0.05 and 0.2, respectively. (TIF) [file ppat.1012069.s001.TIF]

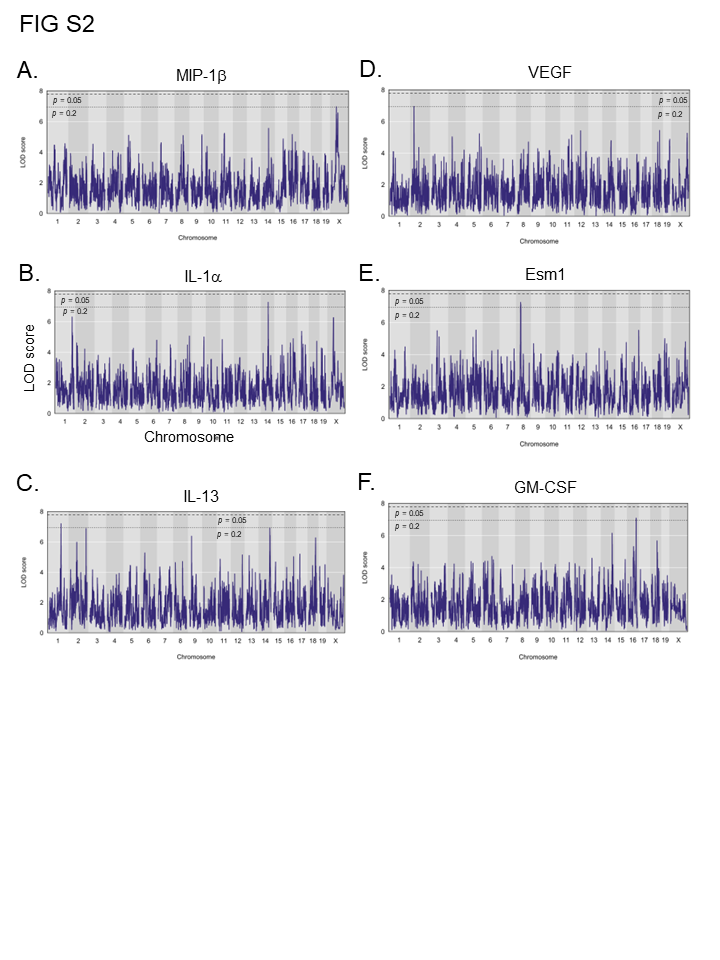

Supplement: S2 Fig — Data from 37 cytokines and chemokines, derived from 300 BCG-vaccinated/M.tb. challenged DO mice, were used to perform QTL mapping. Nine cytokines had significant or suggestive QTL, of which six are presented here: A) MIP-1b, B) IL-1a, C) IL-13, D) VEGF, E) Esm1, and F) GM-CSF. A threshold of p < 0.05 for significant (dashed line) or p < 0.2 suggestive (dotted line) traits was set for the analyses. (TIF) [file ppat.1012069.s002.TIF]

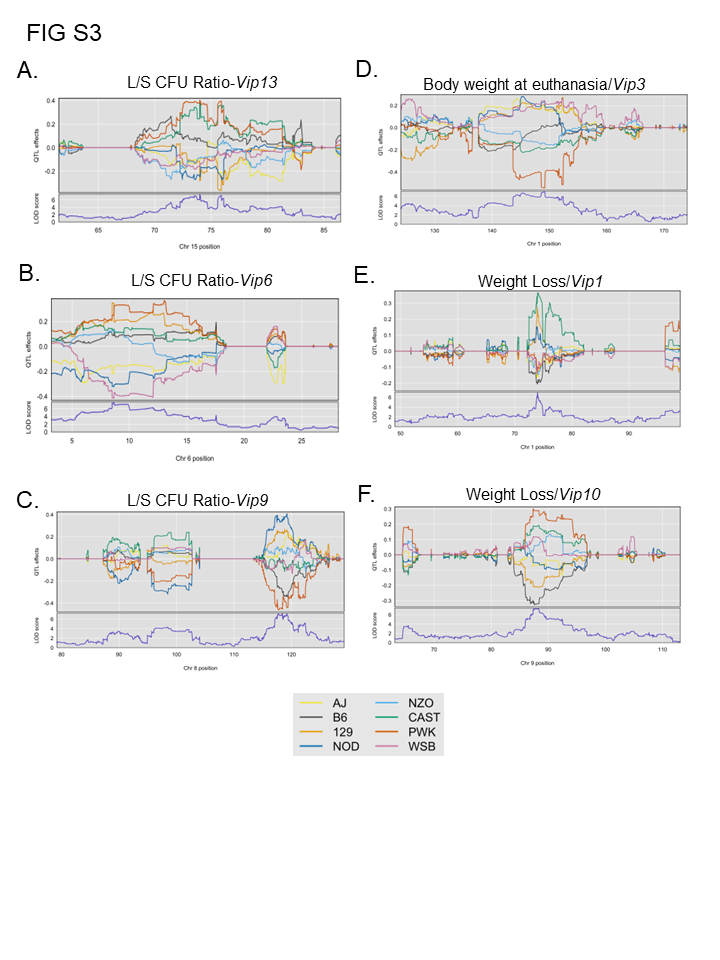

Supplement: S3 Fig — Allele effect plots were generated using the plotting functions of the qtl2 package. Each colored line represents the allelic contribution of a given founder strain, as depicted in the legend. The allele effects were determined for A) Lung/Spleen CFU ratio/Vip13, B) Lung/Spleen CFU ratio/Vip6, C) Lung/Spleen CFU ratio/Vip9, D) Body weight at euthanasia/Vip3, E) Weight loss/Vip1, F) Weight loss/Vip10. (TIF) [file ppat.1012069.s003.TIF]

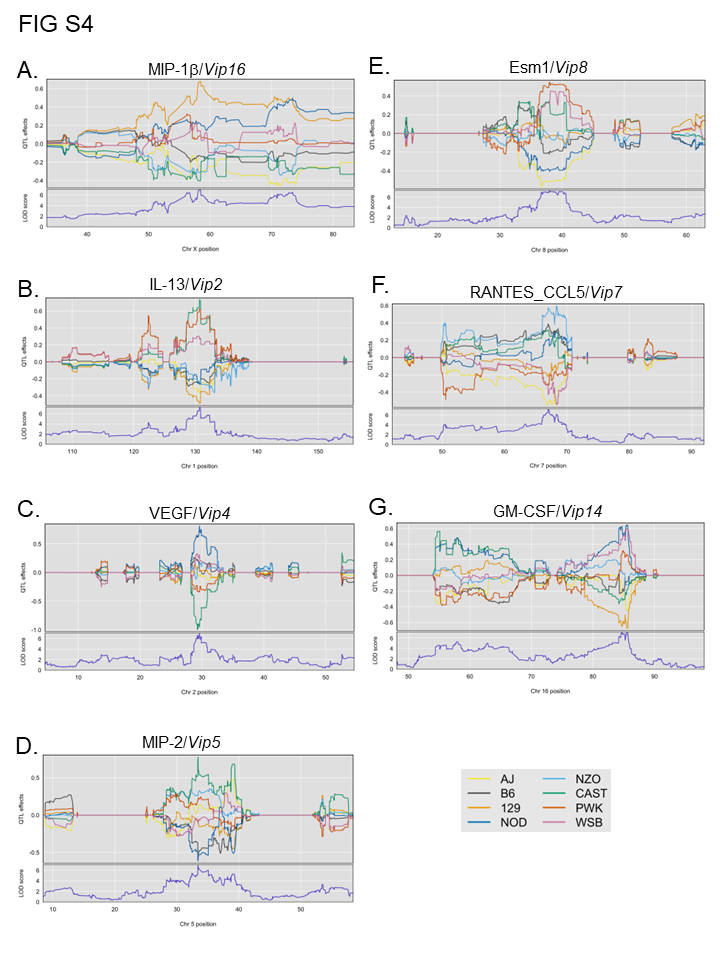

Supplement: S4 Fig — Allele effect plots were generated using the plotting functions of the qtl2 package. Each colored line represents the allelic contribution of a given founder strain as depicted in the legend. The allele effects were determined for A) MIP-1β/Vip16, B) IL-13/Vip2, C) VEGF/Vip4, D) MIP-2/Vip5, E) Esm1/Vip8, F) RANTES/CCL5/Vip7, G) GM-CSF/Vip14. (TIF) [file ppat.1012069.s004.TIF]
